# Synergistic antimicrobial activity between pentacyclic triterpenoids and antibiotics against *Staphylococcus aureus *strains

**DOI:** 10.1186/1476-0711-10-25

**Published:** 2011-06-09

**Authors:** Pooi Yin Chung, Parasakthi Navaratnam, Lip Yong Chung

**Affiliations:** 1 School of Medicine and Health Sciences, Monash University, Sunway Campus, Malaysia; 2 Department of Pharmacy, Faculty of Medicine, University of Malaya, Kuala Lumpur, Malaysia

## Abstract

**Background:**

There has been considerable effort to discover plant-derived antibacterials against methicillin-resistant strains of *Staphylococcus aureus *(MRSA) which have developed resistance to most existing antibiotics, including the last line of defence, vancomycin. Pentacyclic triterpenoid, a biologically diverse plant-derived natural product, has been reported to show anti-staphylococcal activities. The objective of this study is to evaluate the interaction between three pentacyclic triterpenoid and standard antibiotics (methicillin and vancomycin) against reference strains of *Staphylococcus aureus*.

**Methods and Results:**

The activity of the standard antibiotics and compounds on reference methicillin-sensitive and resistant strains of *S. aureus *were determined using the macrodilution broth method. The minimum inhibitory concentration (MIC) of the compounds was compared with that of the standard antibiotics. The interaction between any two antimicrobial agents was estimated by calculating the fractional inhibitory concentration (FIC index) of the combination. The various combinations of antibiotics and compounds reduced the MIC to a range of 0.05 to 50%.

**Conclusion:**

Pentacyclic triterpenoids have shown anti-staphylococcal activities and although individually weaker than common antibiotics produced from bacteria and fungi, synergistically these compounds may use different mechanism of action or pathways to exert their antimicrobial effects, as implicated in the lowered MICs. Therefore, the use of current antibiotics could be maintained in their combination with plant-derived antibacterial agents as a therapeutic option in the treatment of *S. aureus *infections.

## Background

The wide use of antibiotics in the treatment of bacterial infections has led to the emergence and spread of resistant strains. *Staphylococcus aureus *is an important pathogen both in community acquired and healthcare associated infections. The organism has successfully evolved numerous strategies for resisting the action to practically all antibiotics [1]. Resistance to methicillin is now widely described in the community setting (CMRSA), thus the development of new drugs or alternative therapies is urgently necessary.

Plants are known to produce a variety of compounds to protect themselves against a range of microorganisms including plant pathogens and environmental organisms, an indication of the successful defense mechanisms developed. Therefore, plants and their secondary metabolites are a promising source to provide structurally diverse bioactive compounds as potentially therapeutic agents, including antimicrobials. However, plant-derived antimicrobials are less potent. Hence, it becomes apparent that plants adopt a different paradigm - synergy - to combat infections [2]. Synergism has been defined as a phenomenon in which two different compounds are combined to enhance their individual activity. If the combination results in worsening effect, it is called antagonism. Effect which is less than synergistic but not antagonistic is termed as additive or indifference [3].

**Figure 1:**
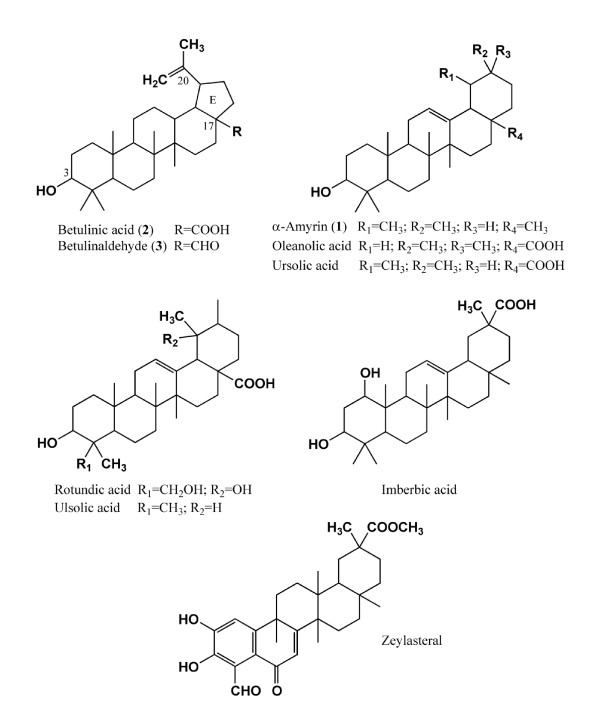
**Pentacyclic triterpenoids isolated and reported to possess antimicrobial activity**.

Antibacterial natural products can be classified according to a general biogenetic source, such as terpenoids, alkaloids, flavonoids and simple phenols. One of the classes with the most active compounds is the triterpenoids, which comprises different types of compounds which may be divided into more important chemical structure groups. The main groups of triterpenoids are represented by tetracyclic and pentacyclic derivatives. Pentacyclic triterpenoids are all based on a 30-carbon skeleton comprising five six-membered rings (ursanes and lanostanes) or four six-membered rings and one five-membered ring (lupanes and hopanes) [4]. Pentacyclic triterpenoids α-amyrin, betulinic acid and betulinaldehyde, and other related triterpenes such as imberbic acid, oleanolic acid (oleanic acid), ursolic acid, ulsolic acid, rotundic acid and zeylasteral have been reported to possess antimicrobial activity (Figure 1) [5-9]. A preliminary study on the antimicrobial activity of α-amyrin, betulinic acid and betulinaldehyde against clinical isolates of MRSA and MSSA showed inhibition at concentrations in the range of 8 to 32 μg/ml [10].

The aims of the present investigation were to assess the antimicrobial activity of the pentacyclic triterpenoids and compare them to the effect of antibiotics (methicillin and vancomycin) on the growth of two reference strains of *S. aureus*; and to evaluate the interaction of the pentacyclic triterpenoids and antibiotics on these strains.

## Methods

### Study compounds and standard antibiotics

The pentacyclic triterpenoids, namely α-amyrin, betulinic acid and betulinaldehyde used in this study were obtained from Sigma Aldrich (USA) and Advanced Technology Co (Hong Kong). The antibiotics methicillin and vancomycin in powder form were also obtained from Sigma Aldrich (USA), each with a potency of 850 μg/mg and 1107 μg/mg, respectively.

### Bacterial strains

The bacterial strains used were *S. aureus *ATCC 29213 and ATCC 43300, which represent methicillin-sensitive and resistant strains, respectively. The bacterial strains were cultured onto Nutrient Agar (NA) (BD Difco, USA) and incubated at 37°C for 16 to 18 hours. Isolated colonies were selected and inoculated into cation-adjusted Mueller-Hinton broth (CAMHB) (BD Difco, USA) to a turbidity comparable to that of 0.5 McFarland standard, which is equivalent to a bacterial count of approximately 10^6 ^cfu/ml.

### Determination of minimum inhibitory concentration (MIC) and minimum bactericidal concentration (MBC)

The three compounds were screened for antimicrobial activity using the macrodilution method [11]. To determine the minimum inhibitory concentrations (MICs), the compounds were dissolved in methanol to give a stock concentration of 10240 μg/ml while the antibiotics were dissolved in ultrapure water to give stock concentrations of 5120 μg/ml. All stock concentrations of compounds and antibiotics were filter-sterilized using 0.20 μm syringe filter. Twofold serial dilutions of the antibiotics and compounds were made with CAMHB to give concentrations ranging from 1 to 512 μg/ml. Five hundred microliter of 10^6 ^cfu/ml bacterial suspension was added to the sterile capped 13 × 100 mm test tubes, giving an inoculum of 5 × 10^5 ^cfu/ml. Another 500 μl of antibiotics or compounds or combinations of antibiotics and compounds were pipetted into the tubes. The control tube did not have any antibiotics or test compounds, but contained the test bacteria and the solvent used to dissolve the antibiotics and compounds. The solvent was shown not to affect the growth of the bacteria during the experiments. The tubes were then incubated at 37°C for 18 to 24 h. The lowest concentration of compound and antibiotic showing no visible growth was recorded as the minimum inhibitory concentration (MIC). Each assay was carried out in triplicate.

From each tube which showed no visible growth, 0.1 ml was subcultured on Blood Agar (BA) (Isolab, Malaysia) to determine the minimum bactericidal inhibition (MBC), which is defined as the lowest concentrations of test compounds that reduced the inoculums by ≥ 99.9%. The plates were then incubated at 37°C for 24 h, after which the colonies were counted. The concentration which has colony count of less than 10 was considered as the MBC.

### Determination of interaction between compounds and antibiotics

Combinations of compounds and antibiotics were tested by the checkerboard method against the two reference strains of *S. aureus*, grown in CAMHB [12]. The concentrations of α-amyrin, betulinic acid and betulinaldehyde tested ranged from 0.25 to 64 μg/ml and for methicillin and vancomycin from 0.25 to 8 μg/ml. The combination for each reference strain was tested in triplicates. The concentration of the individual compound in the combination of pentacyclic triterpenoids and antibiotics in which the growth of organisms is completely inhibited is taken as the MIC of the individual compound in the combination. The fractional inhibitory concentration was calculated as follows:

FIC of compound **a** (FIC_a_) = MIC of compound **a** in combination/MIC of compound **a** alone

FIC of compound **b** (FIC_b_) = MIC of compound **b** in combination/MIC of compound **b** alone

The sum of fractional inhibitory concentration (FIC_s_) indices of two compounds in the combination was calculated as follows: FIC_a _+ FIC_b _= FIC_s_

Synergism has traditionally been defined as an FIC index of 0.5 or less, additivity as a FIC index of more than 0.5 and less than 4, and antagonism as FIC index of more than 4 [13,14].

## Results and discussion

### Antibacterial activity of pentacyclic tritepenoids and antibiotics against reference strains of *S. aureus*

The preliminary study showed that the pentacyclic triterpenoids showed weak antibacterial activity against the reference strains of methicillin-resistant (ATCC 43300) and methicillin-sensitive *S. aureus *(ATCC 29213). Their MICs were in the range of 64 to 512 μg/ml, which is higher than that of vancomycin and methicillin (Table 1). All three triterpenoids exhibited bacteriostatic effect against the reference strains of *S. aureus *at the concentrations tested.

**Table 1:** Minimum inhibitory concentration (MIC) and minimum bactericidal concentration (MBC) values of α-amyrin, betulinic acid and betulinaldehyde on *Staphylococcus aureus *strains

Compounds	MIC (μg/ml)		MBC (μg/ml)	
	
	ATCC 43300^*^	**ATCC 29213**ǂ	ATCC 43300^*^	**ATCC 29213**ǂ
α-amyrin	64	64	> 512	> 512
Betulinic acid	64	64	> 512	> 512
Betulinaldehyde	512	512	> 512	> 512
Vancomycin	4	2	64	32
Methicillin	16	2	> 512	32

* Reference methicillin-resistant *S. aureus *strain

ǂ Reference methicillin-sensitive *S. aureus *strain

### Antibacterial activity of pentacyclic triterpenoids in combination with antibiotics

Based on the FIC calculations (Table 2), the combinations of two compounds involving α-amyrin and betulinic acid, showed synergistic effect with FIC index of 0.5 or less. Betulinaldehyde had synergistic effect only against reference strain of MSSA when in combination with methicillin.

**Table 2:** Susceptibility of reference *Staphylococcus aureus *strains to combinations of α-amyrin, betulinic acid and betulinaldehyde, and antibiotics vancomycin and methicillin

COMPOUND		ATCC 43300					ATCC 29213				
**a**	**b**	^*^MIC_**a**_	^*^MIC_**b**_	^**^FIC_**a**_	^**^FIC_**b**_	^**δ**^**FIC**_**S**_	^*^MIC_**a**_	^*^MIC_**b**_	^**^FIC_**a**_	^**^FIC_**b**_	^**δ**^**FIC**_**S**_

AM	BA	8	8	0.13	0.13	0.26	8	8	0.13	0.13	0.26
AM	BE	-	-	-	-	-	-	-	-	-	-
AM	ME	1	1	0.02	0.06	0.08	1	0.5	0.02	0.25	0.27
AM	VA	1	0.5	0.02	0.25	0.27	8	0.5	0.13	0.25	0.38
BA	BE	-	-	-	-	-	-	-	-	-	-
BA	ME	0.25	4	0	0.25	0.25	0.25	1	0	0.5	0.5
BA	VA	0.25	2	0	0.5	0.5	1	1	0.01	0.5	0.51
BE	ME	-	-	-	-	-	0.25	0.25	0	0.13	0.13
BE	VA	0.5	2	0	0.5	0.5	2	1	0	0.5	0.5

^δ ^AM = α-amyrin, BA = betulinic acid, BE = betulinaldehyde, ME = methicillin, VA = vancomycin; * = minimum inhibitory concentration for combination of two or three compounds expressed in μg/ml as mean of three replicates; ** = fractional inhibitory concentration of individual compounds; ^δ ^= the sum of fractional inhibitory concentration of two compounds in the combination, - = no significant effect on inhibition of growth with the concentrations tested

The MIC for betulinic acid was reduced to 1/8× MIC in the presence of α-amyrin compared to the compound's individual effect (see Tables 1 and 2). Synergy was evident for betulinic acid in combination with methicillin and vancomycin, as the MIC of the compound in the combinations were reduced to 1/64× MIC and 1/8× MIC, respectively. Betulinaldehyde did not show synergistic effect in combinations with α-amyrin, betulinic acid and vancomycin. However, in the presence of vancomycin, the MIC of betulinaldehyde was reduced to 1/1024× MIC against reference strain of MRSA and 1/256× MIC against reference strains of MSSA. Similarly, α-amyrin and betulinaldehyde had a significant effect on enhancing the antibacterial activity of methicillin when tested against the reference strains of MRSA and MSSA, respectively. The combination of the three triterpenoids was also tested, but there was no inhibition on the growth of the organisms at the concentrations tested.

Combined antibiotic therapy may produce synergistic effects in the treatment of bacterial infection and has been shown to delay the emergence of antimicrobial resistance [15,16]. Previous *in vitro *studies have reported synergistic effect of combinations of different plant-derived pure compounds, such as baicalin, tellimagrandin I and rugosin B, and known antibiotic β-lactams against MRSA strains [13,17-19]. Epigallocatechin-gallate (EGCg), a principal constituent of tea, exhibited synergism with antibiotics by destroying β-lactamase activity as well as acting on the peptidoglycan of the cell wall [2]. Berberine, a hydrophobic alkaloid produced by berberry plants, exhibits weak antibacterial properties because of its efflux by multidrug resistance pumps (MDR) in pathogens. When berberine is combined with 5'-methoxyhydnocarpin, also produced by berberry plants, the combination is a potent antibacterial agent as 5'-methoxyhydnocarpin blocks the MDR pumps [20,21]. Synergistic effect between plant-derived compounds and antibiotics enables the use of the respective antibiotics when their effectiveness as single agents are reduced [22]. However, none of the plant-derived compounds, including triterpenoids have successfully been developed for clinical use as antibacterials, probably due to the preference to utilize combinatorial chemistry libraries as a source of chemical diversity rather than natural products [23].

There are numerous reports in the literature on the antibacterial activities of the pentacyclic triterpenoids used in this study. Betulinic acid isolated from the leaves of *Vitex negundo *exhibited antimicrobial activity against *Bacillus subtilis *[24] and *Staphylococcus epidermidis *(MIC of 128 μg/ml). In the same study, no activity was found against the Gram negative bacteria *Pseudomonas aeruginosa *and *Escherichia coli*. Amyrins isolated from *Diopsyros melanoxylon *were found to exhibit antimicrobial activity against both Gram negative and Gram positive bacteria, including *S*. *aureus *(MIC of 90 μg/ml) [5]. α-amyrin obtained from *Trichodesma amplexicaule *Roth. showed activity in both bacteria and fungi, including *S*. *aureus *[25]. Betulinaldehyde isolated from *Diospyros rhodocalyx *exhibited both antimalarial (IC_50 _of 6.25 μg/ml on *Plasmodium falciparum*) and antimycobacterial activity (MIC of 25 μg/ml on *Mycobacterium tuberculosis *H_37_Ra) [26]. In this study, all three pentacyclic triterpenoids exhibited antimicrobial activity against the two reference strains of *S*. *aureus*, one of which was methicillin-sensitive and the other methicillin-resistant. Of the three compounds tested, betulinaldehyde exhibited the lowest effect on *S. aureus *as seen in the high MIC, compared to α-amyrin and betulinic acid. As the structures of these compounds are very similar, their partition coefficients will be similar. Therefore, the differences in MIC, particularly that of betulinaldehyde could be possibly due to the differences in the mechanism of actions of these compounds on *S. aureus *at the cellular level.

Methicillin and vancomycin, agents of β-lactam and glycopeptides classes, respectively, are inhibitors of cell wall biosynthesis. β-lactams block enzymes involved in the cell wall synthesis while glycopeptides block the transglycosylation and transpeptidation reaction necessary to add new subunits to the growing peptidoglycan chain [27]. As the structures of the pentacyclic triterpenoids are different from both these anti-staphylococcal agents, the pathways in the antimicrobial activity of these compounds may have a novel mechanism or target in *S*. *aureus*. The triterpenoids in combination with methicillin or vancomycin could act on different target sites of the bacteria that could theoretically lead to either an additive or a synergistic effect.

However, the mechanism of action of triterpenoids is not fully understood. It has been postulated that zeylasteral and demethylzeylasteral, triterpenoids which exhibited antimicrobial activity against Gram-positive bacteria, block cell division by inhibiting DNA synthesis and macromolecular synthesis in *Bacillus subtilis*. The inhibition of macromolecular synthesis could be due to the damage to the cell membrane [6].

## Conclusion

Synergism against the two reference strains was reproducibly observed between the three compounds and cell wall inhibitors of β-lactam and glycopeptide classes. The best synergistic combination was betulinic acid and methicillin.

## Competing interests

The authors declare that they have no competing interests.

## Authors' contributions

PYC carried out the experimental work and drafted the manuscript. PN and LYC participated in the experimental design, coordination of the study, review of results and draft of the manuscript. All authors have read and approved the final manuscript.
